# Counteracting climate denial: A systematic review

**DOI:** 10.1177/09636625231223425

**Published:** 2024-01-20

**Authors:** Laila Mendy, Mikael Karlsson, Daniel Lindvall

**Affiliations:** Uppsala University, Sweden

**Keywords:** climate change, denial, public understanding of science, science attitudes and perceptions

## Abstract

Despite scientific consensus on climate change, climate denial is still widespread. While much research has characterised climate denial, comparatively fewer studies have systematically examined how to counteract it. This review fills this gap by exploring the research about counteracting climate denial, the effectiveness and the intentions behind intervention. Through a systematic selection and analysis of 65 scientific articles, this review finds multiple intervention forms, including education, message framing and inoculation. The intentions of intervening range from changing understanding of climate science, science advocacy, influencing mitigation attitudes and counteracting vested industry. A number of divergent findings emerge: whether to separate science from policy; the disputed effects of emotions and the longitudinal impacts of interventions. The review offers guiding questions for those interested in counteracting denialism, the answers to which indicate particular strategies: identify the form of climate denial; consider the purpose of intervention and recognise one’s relationship to their audiences.

## 1. Introduction

Despite scientific consensus on climate change reflected by the Intergovernmental Panel on Climate Change (IPCC) (Masson-Delmotte et al., 2021), climate science denial is still widespread (Hornsey and Lewandowsky, 2022) and upheld by organisations motivated by economic and political interests (Brulle, 2014; Oreskes and Conway, 2010a). False narratives are pushed by individuals, think tanks and media outlets, repeated by policy makers (Franta, 2022; Harvey et al., 2018) and targeted towards citizens, whose legitimate concerns about government interventions can be weaponised, to reduce public understanding and support for mitigation policies (Lewandowsky, 2021a) and delay achievement of climate targets (Karlsson and Gilek, 2020). While much research has characterised and explained resistance to environmental science as a whole, climate science denial is found to be the most pervasive issue (Björnberg et al., 2017). Despite much academic focus on the issue, comparatively few studies have systematically examined counteractive interventions and their effectiveness (Björnberg et al., 2017). Considering further the multiple harms that climate denial can have on society, the literature seldom contextualises the intended aims of counteraction in this way.

This review article explores what the scientific literature concludes about counteracting climate science denial, based on a systematic review of journal publications. The objective is to elaborate upon strategies to counteract different forms of climate science denial, to inform scientists, policymakers and others encountering such misrepresentation. We also aim to disclose the reasons behind proposing counteractive strategies, which has not been done before in the literature. More specifically, we answer the following research questions:

- What counteraction interventions of climate science denial are described in the scientific literature?
- What does the literature say about the effectiveness of such interventions?
- What are the intentions of the various types of conducted or proposed interventions being studied?

Next follows a background on the research about climate science denial and a section on the material and method of this review. The results are subsequently presented in a description of proposed interventions and the empirical support for these, and an analysis of the underlying intentions. The final section discusses the results and identifies research gaps.

## 2. Climate denial

In this article, we use the terms ‘climate science denial’ and ‘climate denial’ as labels for a cluster of terms in the literature in combination with ‘climate’ or ‘climate science’, such as denial, doubt and scepticism. This label has been considered polarising (Van Rensburg, 2015) but several enquiries argue that climate science denial is a form of pseudoscience, suffering from ‘a severe lack of reliability’, while being presented as the most reliable knowledge on the issue in question (Hansson, 2017). We therefore agree with those who argue that the alternative term ‘scepticism’ confuses pseudo-scepticism with scientific scepticism (Busch, 2021; Torcello, 2016).

Theories of denial of human atrocities establish the difference between literal, interpretive and implicatory denial (Cohen, 2001). Lifted into the climate context by Norgaard (2006b), it can be argued that *literal denial* is the idea that global warming is not happening; *interpretive* is the acknowledgement of a changing climate but denial of this being a crisis; and *implicatory* is the denial of what can be inferred from the issue in terms of ‘psychological, political or moral implications’ (Cohen, 2001: 8). Another spectrum is trend-attribution-impact denial (Rahmstorf, 2005), all constituting parts of Cohen’s (2001) literal denial. Here, *trend* denial is attached to the global warming phenomenon, *attribution* to the anthropogenic causes and *impact* to the negative effects. Capstick and Pidgeon (2014) explored scepticism in the British public and distinguished between *epistemic denial*, that is, denial of climate science, and *response denial*, that is, denial of the necessity of responding to climate change, echoing Cohen’s (2001) implicatory denial. These clarifications are important, as they describe varying focus and reasons for denial.

Multiple methods and actors spread climate denial, including right-wing media, politicians, religious organisations, vested-interest industry, as well as the general public (Björnberg et al., 2017; Lewandowsky, 2021a). Oreskes and Conway (2010b) explain that people are confused about climate science because they have been deliberately misled. Multiple publications provide evidence of the broad spread of disinformation through, for example, think tanks using false experts to exaggerate mitigation costs, subsequently referenced by politicians (Franta, 2022), fake alternatives to scientific consensus bodies (Medimorec and Pennycook, 2015; Taylor-Neu, 2020) and attacks on social movements in Europe (Moreno et al., 2022). Other studies explore misinformation on social media, for example, through twitter bots (Marlow et al., 2021).

The susceptibility to believe misrepresentations is also explored. Multiple studies focus on worldviews of climate science denying actors and find multiple motivations and predictors such as libertarian ideologies (Lewandowsky, 2021a), political identities (Feygina et al., 2010; Whitmarsh and Corner, 2017), religious evangelism (Edvardsson Björnberg and Karlsson, 2022), anti-feminist and anti-immigrant values (Jylhä et al., 2020) and social construction and emotional protections (Norgaard, 2006a). Much of this literature addresses the political implications, particularly in terms of rejection of climate policy.

The focus of the literature has been on understanding causes and characteristics of climate science denial, but ‘the scientific community ignored the question of how to effectively counter arguments of science denialism for too long’, even though leaving denial unaddressed can result in further negative attitudes (Schmid and Betsch, 2019: 931). Hornsey and Lewandowsky (2022) thus offer six strategies for reducing the damage of climate scepticism, both ‘before and after misinformation takes seed in people’s minds’. These studies are recent answers to calls for ‘more research. . . that focuses on assessing, developing and comparing speciﬁc strategies to counter science denial’ (Björnberg et al., 2017: 239). The present article further responds to this knowledge gap.

## 3. Materials and method

This study follows the process of a systematic review, aiming for a transparent, non-biased selection and analysis of literature corresponding to the objective and research questions described above (Denyer and Tranfield, 2009). The study is limited to peer-reviewed articles in scientific journals up to year-end 2021, found through applying the following Scopus search string:

(TITLE-ABS-KEY ((denial* OR deny OR skeptic* OR doubt* OR disinform* OR misinform*) AND (scien* OR evidence OR *inform*)) AND TITLE-ABS-KEY (climate OR ‘global warming’)) AND PUBYEAR < 2022 AND (LIMIT-TO (SRCTYPE, ‘j’)) AND (LIMIT-TO (DOCTYPE, ‘ar’) OR LIMIT-TO (DOCTYPE, ‘re’)) AND (LIMIT-TO (LANGUAGE, ‘English’))

These terms intend to capture all literature focused on climate science denial and the search resulted in 1354 hits. A selection process was carried out, guided by a protocol (Supplement A), resulting in a final list of 65 articles. Given the breadth of research interest in the issue of climate science denial, we do not presume that the search terms necessarily result in identifying all relevant articles. The selection may have led to overlooking some relevant articles, but we are nevertheless confident to cover, with margin, the core of the relevant area.

The selected 65 articles were analysed based on a series of topics linked to the research questions, mainly concerned with (1) the form of climate science denial in focus and the counteracting strategy proposed, (2) the effectiveness of the strategy and (3) the intentions behind interventions.

## 4. Results

More than half of the selected articles are published in 2018–2021, and the first one came just 10 years earlier (McCaffrey and Buhr, 2008). Spanning 44 journals, the publications have no obvious ‘home journal’, though are typically found in journals of social sciences and psychology as well as environmental and earth sciences. Cook, Lewandowsky, Feygina and Hansson are the most common authors, with Feygina et al. (2010), Cook et al. (2017) and Bain et al. (2012) being the three most cited articles. There is furthermore a clear US focus, followed by Australia and the United Kingdom, in the retrieved material.

Of the 65 articles, 27 are empirical studies that test different interventions and explore the measured impact on attitudes, political preferences and personal behaviour intentions. Tables 1 and 2 provide overviews of these studies. Twenty-seven studies are reviews, overviews and perspectives. In these, counteractions are asserted without necessarily providing empirical evidence of their impact. In addition, 11 articles make claims towards promising counteractions based upon empirical studies. See Supplement B for a full list of the articles in review.

**Table 1.** Epistemic denial forms and the strategies explored in empirical studies.

	Strategies	Outcomes and impacts	Weaknesses (in strategy and methods of study)
GBM	Knowledge dissemination	Reduces denial, increases political action support	Motivated reasoning challenging, pre-tests sensitise participant; inconsistent results
	Elite cue correction	Republican decision-maker effectively corrects misperceptions in participants of all political backgrounds	Democratic decision-maker not effective, conservatives remain least likely to believe in climate change
	Content-based Inoculation	Inoculation immediately prior to misinformation increases perception; resistance to misinformation lasts up to 1 week	Specific to issue only; unclear whether this can be attributed to inoculation or scientific consensus messaging
	Logic-based inoculation	Neutralises effect, increases beliefs, policy support	
Trend	Message framing	Localising impacts increases beliefs; empathic messages increases acceptance; free-market frames can increase conservatives’ beliefs; national security and economic frames of emissions reductions neutralises the effect of denial statements	Informative, persuasive frames limited; religious frames do not significantly affect conservatives’ beliefs; free-market frames increase beliefs only in conservatives
	Co-benefits	Mitigation co-benefits in response to misinformation leads to more engagement and agreement on social media.	
	Correction	Can neutralise presidential falsehoods, lead to more factual accuracy; does not necessarily backfire	Correction of presidential misinformation does not increase wider beliefs in climate change
Attribution	Critical thinking	Focus on logic flaws reduces denialist claim influence, increases beliefs, increases pro-environmental behaviour intentions; leveraging cognitive consistency of trusting other science increases stated beliefs	Study methods may have sensitised participants
	Message framing	Frames that explain the mechanism in greenhouse gas effect more effective in increasing attribution beliefs than risk frames	Relies upon an open-minded attitude
	Co-benefits of behaviour	Community care, economic and technological development co-benefits increase pro-environmental behavioural intentions	Avoids communicating mitigation benefits, thus may not increase beliefs in climate change science
Impact	Certainty range	Increases trust in projections and concern about climate change	Not assessed in terms of general climate change beliefs
	Deliberation	Deliberation, impact scenarios reduces strength, type of scepticism	A year later sceptical participants return to original discourse position
	Visualisation of hazards	Increases acceptance of climate science and political interventions, effective across political spectrum	

GBM: gateway belief model.

**Table 2.** Counteractions in relation to response denial found in the empirical studies.

	Outcomes and impacts	Weaknesses
Trusted elite cues	Conservative politicians, military sources increase political support in students; evangelical climate change scientist increases beliefs in evangelical students; military cues increase concern and beliefs	May not necessarily increase support for political action
Message Frames	Negative emotional experience increases climate change concern, mitigation support; nature videos with mixed emotional tones are effective in increasing environmental concern; preservation of socio-political system frames increases pro-environmental behavioural intentions	Support for mitigation may pre-exist for other reasons, these methods are vulnerable to exposure bias
Education	Increases belief in conservatives; overcomes scepticism in adolescents with individualistic worldviews; reduces denialism in those with high levels of social dominance orientation	Selective exposure bias could deter conservatives from climate change education and information

The next section accounts for the different strategies explored within the literature to counteract climate science denial (henceforth labelled ‘climate denial’). Thereafter, empirical studies with evidence for the effectiveness on different forms of climate denial are extracted. Finally, the intentions within the counteraction of climate denial are categorised.

### Counteractive responses to climate denial

This section accounts for all counteraction interventions explored within the 65 reviewed articles, as well as the theoretical underpinnings supporting different strategies.

#### Understanding the form of denial is central for the counteraction

We found 13 review and overview papers that discuss multiple counteraction strategies in relation to multiple forms of denial, scepticism or wider climate attitudes. Three of these emphasise the importance of understanding peoples’ positions as fundamental to the different messaging and communication strategies (Hornsey and Fielding, 2017; Rode et al., 2021; Van Rensburg, 2015). A meta-analysis exploring methods for changing all forms of climate attitudes in the United States found that the ‘type of climate change attitude matters more than the type of intervention’ (Rode et al., 2021). This approach was repeated in terms of ‘jiu-jitsu’, the martial art that uses an attacker’s own moves against them; understanding the roots of an attitude is central to change it (Hornsey and Fielding, 2017). Van Rensburg (2015) explain in two taxonomies of climate scepticism that the decision to intervene to change an attitude should be based upon understanding how strongly one holds those attitudes, and whether it is directed towards climate science, the knowledge-making process or the responses to climate change.

An alternative categorisation of strategies is based on the time of exposure to misinformation (Treen et al., 2020). During pre-exposure to climate misinformation, education and inoculation are suggested. Inoculation appears successful to reduce the effects of misinformation (Compton et al., 2021; Cook et al., 2017; Lewandowsky, 2021b). Technological solutions are proposed, including detection of bots and adjusting social media algorithms. Post-misinformation, factual correction and punitive measures are suggested. The categorisation by Treen et al. (2020) resonates with another review (Cook, 2017), which discusses how strategies produced post-exposure had limited success: debunking is less effective in dislodging misinformation and may lead to backfire if conflicting with worldviews.

In the remaining review papers, multiple counteractions are detailed or clustered in a manner that echoes the categorisations described by Björnberg et al. (2017): ‘the need for change’ in academic messaging, ‘the need for context-dependent strategies’, ‘communication strategies’, ‘education’ and ‘changing the focus of scientists’. These include, among other techniques, improving education (Das, 2020; Goodwin and Dahlstrom, 2014; McCaffrey and Buhr, 2008; Ranney and Velautham, 2021), message framing (Wong-Parodi and Feygina, 2020), inoculation against misinformation (Compton et al., 2021), appeals to conservative values and policies that favour the status quo (Hornsey and Fielding, 2017), and emphasising scientific consensus (Lewandowsky, 2021b; Rode et al., 2021). The latter technique, however, is reported to have mixed effectiveness, despite its popularity (Rode et al., 2021: 11): ‘emotion, psychological distance (near) and religious interventions displayed the most promise’ in changing general attitudes.

#### Message frames can appeal to targeted audiences

Multiple studies explore message framing, a process used to emphasise particular aspects of an issue through language choices to create or enforce different narratives (Busch, 2021). Studies assert the effectiveness of using particular frames for reducing denial by aligning messages to audiences’ cultures (McCright et al., 2016), identities and values (Das, 2020; Dixon et al., 2017; Lo, 2014; Romero-Canyas et al., 2019; Turnpenny, 2012), concerns and risks of hazards (Bolsen et al., 2019; Sterman, 2011; Watts, 2019) or co-benefits of climate mitigation (Lawrence and Estow, 2017). Frames can also target specific climate beliefs: framing a message about climate change mechanisms rather than climate change risks can increase the belief in human attribution (Rotman et al., 2020). Message framing is theoretically linked to the roots, contexts and ideological belief systems through which denial is constructed (Hornsey and Fielding, 2017); appealing to those contexts and belief systems can more effectively reach audiences (Rode et al., 2021; Rudiak-Gould, 2013; Van Rensburg, 2015).

Message framing not only increases audience receptivity to information, it can also affect behaviour change and support for mitigation (Bain et al., 2012; Feygina et al., 2010). Where behaviour change is the aspired outcome, it can be effective to disassociate climate from behaviours to avoid politically contentious issues; communicators can rather assert benefits of behaviour change, for example, for household economy and health (Shirley, 2021). For those sceptical about behaviour change, it may be beneficial to acknowledge a low impact of individual behaviour on the climate while asserting other advantages (Capstick and Pidgeon, 2014).

Four key message frames are proposed to foster climate mitigation: emphasising systems-sanctioned change for those with systems-justification ideologies; relating actions to political and cultural affiliations and identities; removing misperceptions of social norms to demonstrate that action is socially sanctioned; and enabling self-affirmation by allowing people to be open with their beliefs and curating deeper conversation space (Wong-Parodi and Feygina, 2020).

Framing messages to be more or less empathic, informative and persuasive can also reduce denial (Munoz-Carrier et al., 2020). Nature videos with a mixture of both positive and negative tones were more effective in increasing environmental concern, rather than one or neither tone (Franzen and Mader, 2020). Where denial is the emotional protection in face of alarming scientific information, emotionally empowering communication can reduce denialism and increase behaviour change (Haltinner and Sarathchandra, 2018). While some literature argues against using fear in messaging (Das, 2020), some state that negative emotional experience can also increase concern and mitigation support (Wong-Parodi and Feygina, 2021). The contradictory assertions of utilising emotional experiences in communication indicate research gaps.

#### Inoculation reduces the uptake of misinformation

Misinformation can confuse the public and increase climate denial. Inoculation can counteract these causes (Farrell et al., 2019; Lewandowsky, 2021b; Treen et al., 2020; Trémolière and Djeriouat, 2021). The strategies vary in the literature to focus on specific content or logic in misinformation (Cook et al., 2017, 2018). Inoculation is typically studied immediately prior to exposure; however some limited longitudinal effects have been found (Maertens et al., 2020). One review of inoculation explains the method of exposing individuals to a mild form of misinformation to equip people with mental defences without becoming victim to the misinformation within the inoculation (Compton et al., 2021). Active and passive methods exist, respectively where rebuttals are made by participants or provided for them. There are, however, research gaps, particularly with regard to wider spread of inoculation (Compton et al., 2021). It is elsewhere argued that some attribution of the impact of inoculation underplays the factor of consensus messaging within these studies (Williams and Bond, 2020). Earlier literature discussed the study of ignorance, agnotology, in schools as a method to counter climate misinformation (Bedford, 2010).

#### Education and science communication remain central

According to Lewandowsky (2021b: 67), ‘knowledge matters’. Communicating scientific consensus is widely discussed (Benegal and Scruggs, 2018; Björnberg et al., 2017; Chinn and Hart, 2021; Cook, 2017; Dixon et al., 2017; Hansson, 2018; Lewandowsky, 2021b; Maertens et al., 2020; Rode et al., 2021; Romero-Canyas et al., 2019; Sauer et al., 2021; Williams and Bond, 2020). A recent study argues that inconsistent results with consensus messaging may be attributed to sensitising participants through pre-tests in study methods (Chinn and Hart, 2021). Three studies provide evidence for the knowledge deficit thesis, that education can reduce misperceptions and increase beliefs in spite of, though still limited by, different motivated reasoning barriers (Häkkinen and Akrami, 2014; Hess and Maki, 2019; Stevenson et al., 2014). Others found that correcting misinformation could increase factual accuracy, though did not necessarily influence other attitudes towards climate science (Porter et al., 2019; Watts, 2019).

Increasing climate literacy (Busch, 2021; Hansson, 2018; McCaffrey and Buhr, 2008) or social media literacy (Chen et al., 2021) have been proposed. Improved communication methods for the IPCC are asserted based on the premise that low scientific understanding risks denial in the public (Sterman, 2011). Accordingly, accommodating to the skillsets of audiences through reducing complexity, considering enquiry skills, using relatable models and experiential learning techniques is all suggested (Sterman, 2011). Specific pedagogical techniques are mentioned including agnotology (Bedford, 2010), humbling experiences (Ferkany, 2015) and the use of extra-curricular projects and social justice perspectives in climate courses (Ranney and Velautham, 2021). Including images and certainty ranges could also improve science messages (Bolsen et al., 2019; Joslyn and LeClerc, 2016), and more clearly communicating scientific methods (Sarathchandra and Haltinner, 2020). Increasing critical thinking skills and cognitive consistency is suggested (Ferkany, 2015; Gehlbach et al., 2019; Johnson, 2017). Improving educators’ understandings of climate science was also of focus in the literature (Sezen-Barrie et al., 2019).

There is some debate over how scientists, themselves, should increase trust in their science. Public outreach is asserted in a number of studies (Das, 2020; Hansson, 2018; Harvey et al., 2018). Increasing trustworthiness of the scientist is considered a four-step process by Goodwin and Dahlstrom (2014): (1) demonstrating vulnerability in meeting with sceptical audiences, (2) providing an empowering opportunity to assess scientists’ statements, (3) being transparent about previous errors and (4) separating policy from science. It is elsewhere argued that scientists can advocate for the credibility of their colleagues to increase uptake of information (Sarathchandra and Haltinner, 2020). Alternatively, finding trusted leaders for climate communication can reach audiences who otherwise may be alienated (Motta et al., 2021; Sauer et al., 2021; Webb and Hayhoe, 2017).

Lewandowsky et al. (2015) argue that scientists should not allow pseudoscientific thinking to set the agenda, and caution against debating with someone unwilling to change their mind as it can lend legitimacy to a denialist position. This is questioned, however, since unconvinced individuals should not be disregarded prima facie as some, particularly those without vested motivations, may be open to discussion (Van Rensburg and Head, 2017). Considering the trends in post-normal science, such as deliberation and co-production, it is also claimed to be important that scientists recognise a shared influence with other epistemes in policy-making: a scientist should contend with the socio-political underpinnings of science-making in the current era (Turnpenny, 2012). This contradicts the assertions from Goodwin and Dahlstrom (2014) to separate science from policy. An alternative suggestion is to explain the epistemology of science (Mason, 2020). Again, contradictory findings underline research gaps.

#### Meeting conflicting worldviews and deliberation may shift beliefs

Few studies explore deliberation or intergroup relations. These are clustered together here, despite considerable difference in the methods, predominantly due to underlying theories of purposefully meeting with conflicting worldviews. An empirical study finds that denialism is associated with intergroup conflicts and asserts the value of improving intergroup relations (Bliuc et al., 2015). It was found that denialists could temporarily migrate their beliefs across different discourses of denialism through participating in deliberative forums (Hobson and Niemeyer, 2013).

#### Methods to expose motivated agendas are understudied

Some of the reviewed articles focus on those who spread denial. Farrell et al. (2019) propose that coinciding with methods to change beliefs, legal efforts should also be directed towards the economic and political structures of misinformation. Online, climate misinformation can be targeted through detecting malicious accounts or through punitive measures (Treen et al., 2020). It could also be strategic for scientists to reveal the funding of different false experts and the economic interests in sowing doubt (Harvey et al., 2018), not least as it provides to the public a reason for believing that some information might be considered false (Leuschner, 2018). Hansson (2017) summarises that scientists should not:

. . . act as if the denialists’ fake controversies were real controversies, and neither should we accept the deviant criteria of scientiﬁc assent that they try to impose on us. Our task is instead to expose their strategies, their agenda, and the pseudoscientiﬁc characteristics of their argumentation. And above all it is our task to explain what science really is, why it should not be politicized, and how it can provide humanity with a better common understanding of the world we are living in (p. 45).

### Evidence of the effectiveness of counteraction strategies

Twenty-seven of the reviewed articles are empirical studies exploring the effectiveness of counteraction strategies. In line with Capstick and Pidgeon (2014), we describe these in the two broad categories of epistemic and response denial.

#### Reduction of epistemic climate denial

The studies exploring interventions towards epistemic denial can be divided into four types (Table 1): the perception of scientific consensus, as depicted in the so-called gateway belief model (GBM),^1^ and trend, attribution and impact denial (Rahmstorf, 2005). These types are matched with the counteraction strategies found in the reviewed articles.

##### Counteracting misperceptions about the scientific consensus

Several studies attempt to improve factually accurate understanding of scientific consensus, including through knowledge dissemination (Chinn and Hart, 2021), elite cue correction (Benegal and Scruggs, 2018) and content- and logic-based inoculation (Cook et al., 2017; Maertens et al., 2020; Williams and Bond, 2020), all found effective in changing perceptions and beliefs, and neutralising misinformation. However, only one study found results in terms of policy support (Cook et al., 2017). Some weaknesses of this approach are also accounted for, particularly that motivated reasoning continued to bias information reception (Chinn and Hart, 2021). Knowledge dissemination was found inconsistent in changing beliefs and policy support in the wider literature in review (Dixon et al., 2017; Sauer et al., 2021). Conservatives remained the least likely to believe in climate change, despite corrections from a republican decision-maker, though more effective than a democrat (Benegal and Scruggs, 2018). Content based inoculation, here about the scientific consensus, has limited effect (Cook et al., 2017). In addition, effectiveness could be attributed to other aspects under study or methodological limitations (Chinn and Hart, 2021; Williams and Bond, 2020).

##### Counteracting denial of climate change trends

Multiple studies explored how message framing could reduce selective information uptake on trend denial. Empathic messages (Munoz-Carrier et al., 2020), free-market frames (Dixon et al., 2017) and localising impacts (Romero-Canyas et al., 2019) were all found to increase beliefs and acceptance of climate change. Informative and persuasive message frames were found less effective in changing beliefs (Munoz-Carrier et al., 2020). One study shows that emphasising economic benefits and national security of emissions reductions is effective in counteracting denialist statement on individual beliefs, and more effective than using stewardship or public health frames (McCright et al., 2016). Religious frames were found to be ineffective in influencing beliefs in conservatives, and free-market frames were found to be effective only on conservative participants (Dixon et al., 2017). In response to misinformation, using cold weather as proof against global warming, communication of co-benefits was highly regarded and led to increased engagement on social media (Lawrence and Estow, 2017).

Following misinformation spread, correcting president Trump’s false statements on climate change could lead to more factually accurate information in study participants, but did not impact wider climate change beliefs (Porter et al., 2019). Despite the concern of backfire, correcting online misinformation does not lead to increased climate denial (Lawrence and Estow, 2017).

##### Counteracting denial of the anthropogenic cause of climate change

The studies in this group explore two forms of counteraction strategy: increasing critical thinking and communicating co-benefits. Inviting participants to focus on the flawed logic in misinformation resulted in reducing influence of misinformation, increasing overall climate change beliefs and pro-environmental behaviour intentions (Johnson, 2017). A survey study explored leveraging cognitive consistency, through demonstrating that participants trusted other fields of science and, therefore, to remain consistent, reported higher beliefs in climate science (Gehlbach et al., 2019). The effectiveness of framing messages to explain climate change was more pronounced than risk frames, had a lasting effect, and could promote environmental behaviour (Rotman et al., 2020). On co-benefits, one study shows that caring for one’s community, or behaviour that contributes towards economic or technological development, increases pro-environmental behaviour intentions (Bain et al., 2012).

##### Counteracting the denial of climate change impacts

Two studies used scientific communication methods to reduce misperceptions of climate impacts. Visualisations of hazards were shown to increase acceptance of science and support for mitigation policy irrespective of political beliefs and preferences (Bolsen et al., 2019). By including the certainty range of projections, the trust in science increased (Joslyn and LeClerc, 2016). The use of climate scenarios in deliberation forums can temporarily reduce strength and change form of denial (Hobson and Niemeyer, 2013). It was found, however, that those who were interviewed a year later had re-migrated to their original positions.

#### Reduction of response climate denial in empirical studies

Based upon literature that has found higher levels of response denial in conservatives, particularly among Republican voters in the United States, the studies in this category typically explore intervention strategies and then test averages of beliefs. Table 2 accounts for studies specifically exploring the effectiveness of different counteraction strategies on conservative, right-wing or Republican beliefs in relation to response denial.

##### Trusted elite cues

Three studies support the thesis that trusted sources can be effective information transmitters. Considered here as trusted elite cues, it was found that conservative politicians and military could increase reported levels of support for mitigation policy in conservative students (Sauer et al., 2021). Evangelical students increased their climate change beliefs following a lecture by an evangelical climate scientist (Webb and Hayhoe, 2017). Military cues were found to increase levels of concern and beliefs about climate change in conservatives more generally, but unlike with conservative students, this did not increase support for mitigation policy (Motta et al., 2021).

##### Message framing

Using emotional narratives to curate a negative experience in participants increased conservative concern about climate change and support for mitigation (Wong-Parodi and Feygina, 2021). Nature videos increased environmental concern in sceptical research participants when they contained both positive and negative tones (Franzen and Mader, 2020). Messages that reinforce and preserve socio-political systems preferred by conservatives with systems-justification reasoning were found to increase pro-environmental behaviour intentions (Feygina et al., 2010).

##### Education

Arguably all the methods presented in the ‘Reduction of response climate denial in empirical studies’ section relate to education in terms of knowledge dissemination. Education was found to increase belief levels in conservatives (Hess and Maki, 2019) and overcome individualistic worldviews in adolescents (Stevenson et al., 2014). An IPCC newscast was effective in reducing levels of denial in those with social dominance orientation, an ideology that presumes natural social hierarchies (Häkkinen and Akrami, 2014). These studies, however, rely on the willingness of participants to be exposed to information, and selective exposure bias may deter, for example, students taking climate change courses (Hess and Maki, 2019). This suggests a limited effectiveness of all knowledge dissemination techniques.

### Intentions of counteracting climate denial

Counteracting climate denial can be done for different reasons. Here we categorise the intentions of intervention from all 65 reviewed articles. Four types emerged from the systematic review: two relating to science and knowledge and two concerning mitigation and public debate. The first intention is to increase accurate public understanding of climate change science: ‘Targeting Epistemic Beliefs’. The second is ‘Science Advocacy’, related to the responsibilities of the scientist, asserting the value of science to the public. The third, ‘Changing Response Attitudes’, is to increase support for climate mitigation, from personal behaviours, increasing policy support, or otherwise. The fourth is ‘Countering Motivated Denial’, targeted towards vested-interest groups and their activities. The intentions behind the counteraction strategies in the reviewed articles are either explicitly stated or appear implicitly, for example, from the study aim or the framing of climate denial, or in terms of intended effects of interventions.

#### Targeting epistemic beliefs

Most counteraction efforts can be considered to aim for affecting the public understanding of climate science, though deliberative methods and the strategies that expose agendas appear least likely to consider influencing epistemic beliefs. By contrast, inoculating the public against misinformation, and education and scientific communication, overwhelmingly focus on these outcomes.

#### Science advocacy

There are strong assertions within the reviewed articles about the important role of the scientist and of science in society. As such, a few strategies aim to advocate for climate science. Science advocacy stands out as a complex and divisive issue though and there are differing assertions about the separation of science and policy in the public eye.

#### Changing response attitudes

Based upon theorisations that values underpinning policy and behaviour preferences could be swayed, message framing largely focuses on influencing response attitudes. Inoculation and exposing agendas are least likely to consider these outputs. It can be assumed, though, that reducing the capacities of vested interests, or neutralising their spread of disinformation by inoculation, could produce long-term outcomes related to response attitudes and successful climate mitigation.

#### Counteracting motivated denial

The fourth intention is the aspiration to counteract vested-interest groups and their work. Such intentions can be realised through reducing the risk of misperceptions through inoculation. Some studies argue for revealing funding, networks and techniques of denial organisations. Education and scientific communication and message framing were least likely to aim to counteract motivated denial.

#### Linking intentions to strategies

**Figure 1.**
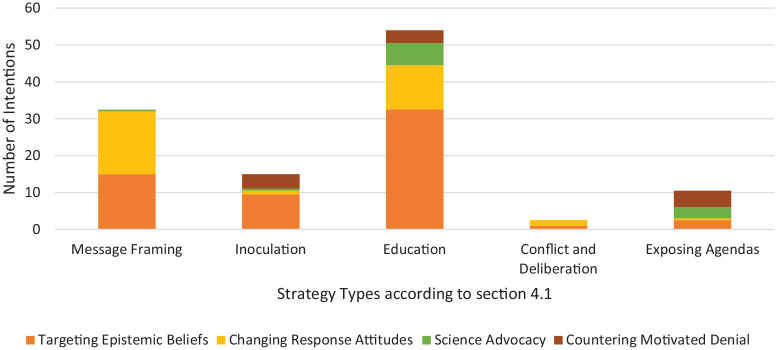
A representation of the multiple intentions for counteraction from all reviewed articles. *Note*: please refer to online version for colour figure.

Figure 1 illustrates how the strategy types described in the ‘Counteractive responses to climate denial’ section had multiple intentions and how common the intentions were in each strategy. A strategy may have more than one intended outcome. Different strategies had different intentions, except for a strong general commitment to foster the public understanding of climate science. The reviewed studies also focus on the reputation of climate change science, albeit message framing strategies appear more interested in influencing response attitudes, for example, support for mitigation policies. For inoculation strategies, counteracting vested interests was the second most common preferred outcome.

## 5. Discussion

The research on climate denial is well established, with extensive documentation of long-standing denial campaigns (Franta, 2022), and detailed analysis of the characteristics and explanations of denialism. This review adds a systematic and comprehensive description and analysis of the research focusing on counteractive interventions to climate denial. It further accounts for the empirical evidence of the effectiveness of various interventions and documents the intentions of counteractions. The many divergent findings identified within the literature indicate significant research gaps.

Our first observation is that the research on ways to counteract climate denial, despite a growing interest in the field since the review by Björnberg et al. (2017), is still limited, and to a large extent still theoretical rather than empirical or focused on the effectiveness of interventions. Recent overviews (Hornsey and Lewandowsky, 2022) provide important contributions and recommendations, but as shown here, results and conclusions still point in different directions on some key issues. Some of this may be attributed to the different drivers of climate denial and the different purposes for intervention. However, it is not clear to what extent this is the case, and more specificity in the intentions could be helpful in future research. A main issue concerns whether to separate science and policy in public, or to acknowledge the interconnectedness of these issues to depolarise responses to climate information. While some studies consider empathising with the roots of climate attitudes, others emphasise the risk of this on diverting scientists’ energies where these attitudes can be illegitimate or pseudoscientific. Furthermore, the effects of using emotional narratives are disputed. More research on these aspects would be welcomed.

A second observation concerns the challenge that change in the public understanding of climate science due to successful interventions, in unchanged social, political or economic systems, may still be ineffective for climate mitigation (Lewandowsky, 2021b). Changing views without providing hopeful opportunities could, namely, lead to a sense of alarm or helplessness in the public, backfiring in terms of a renewed cause of denial for emotional protection (Norgaard, 2006a). Adding that most claims of effectiveness are time and context specific, underlines a considerable research gap. Besides pointing out that counteractive measures ought to be considered in parallel with climate governance and societal dialogue more broadly, additional studies are needed to better understand the long-term and contextual aspects of counteractive interventions. Moreover, since much focus in the reviewed studies is placed on individuals and their beliefs, plausibly due to the overwhelming representation of science communication, sociology and psychological studies within the literature, studies are needed on counteracting measures at the societal level, for example, on legislation for the spread of disinformation.

A third observation is that the reviewed studies seldom explore why intervention efforts of scientists, science communicators, and climate governance actors have often been insufficient. Arguably, by engaging in activities that aim to counteract those who spread denial, these groups run the risk of being considered partisan in a manner principally similar to vested fossil fuel interests being accused. This may explain why several researchers are reluctant to engage with the full spectrum of climate misrepresentations and points towards both research gaps and the necessity for researchers to increasingly reflect on their roles (Pielke, 2007), and how to uphold academic independence and integrity when counteracting climate denial. More research is nevertheless needed to better understand how scholars and experts may engage with climate misrepresentations in public debate, politics and courtrooms.

Evidently, this review shows that no advice can be given on how to universally counteract climate denial. For those interested in doing so, however, three guiding questions can be of value: first, is it epistemic or response denial that is to be engaged with? As our review shows, different strategies are suitable for specific forms of denial, and for specific drivers (see Tables 1 and 2 for a guide). Second, what are the preferred outcomes of a counteraction intervention; is it changing minds, behaviour or policies? If the intention is to increase support for behaviour change or political action (response denial), then message framing strategies might be more helpful than inoculation against misinformation. Our review accounts for the different ways in which strategies have been developed to affect a number of different outcomes. Third, what is one’s own role – for example, a scientist, expert or politician – and how might it be perceived and the intervention be interpreted? In some cases, using other trusted sources to communicate the message may be necessary.

Finally, we assert that much work remains to be done. Despite the IPCC’s most recent assessments, demonstrating the urgency for rapid climate mitigation, the widespread delay of goal achievement, rooted in epistemic but predominantly response denial, persists. As mitigation efforts continue to advance, it is important to understand how to gain enhanced public acceptance. Counteracting denial is critical in that endeavour.

## Supplemental Material

sj-docx-1-pus-10.1177_09636625231223425 – Supplemental material for Counteracting climate denial: A systematic review Supplemental material, sj-docx-1-pus-10.1177_09636625231223425 for Counteracting climate denial: A systematic review by Laila Mendy, Mikael Karlsson and Daniel Lindvall in Public Understanding of Science

## Notes

1. The GBM proposes that understanding the scientific consensus about climate trends and human attribution is an indicator of wider positive attitudes about climate change science (Cook et al., 2017).
