# Supplementary material for: Counteracting climate denial: A systematic review
Source: Public Underst Sci. 2024 Jan 20;33(4):504–20. doi: 10.1177/09636625231223425 (PMC11056086; doi:10.1177/09636625231223425)
Supplement: sj-docx-1-pus-10.1177_09636625231223425 – Supplemental material for Counteracting climate denial: A systematic review [file sj-docx-1-pus-10.1177_09636625231223425.docx]

**Counteracting climate denial: a systematic review**

**SUPPLEMENTAL MATERIAL**

Authors:

Mendy, Laila

[Laila.mendy@geo.uu.se](mailto:Laila.mendy@geo.uu.se)

Corresponding author

Karlsson, Mikael,

[Mikael.karlsson@geo.uu.se](mailto:Mikael.karlsson@geo.uu.se)

Lindvall, Daniel

[Daniel.lindvall@geo.uu.se](mailto:Daniel.lindvall@geo.uu.se)

Contents:

Supplement A: Instruction for Selection of Articles ……………………………………………………………………… 2

Supplement B: List of 65 Articles reviewed within Section 4 ………………………………………………………… 4

**Supplement A: Instruction for Selection of Articles**

The identification of articles to analyse in the review was done in two stages, one before manuscript submission, the second in response to comments from reviewers, with multiple steps. The following accounts for the entire procedure.

**Stage One**

**Step One:** Scopus search string:

( TITLE-ABS-KEY ( ( denial* OR deny OR skeptic* OR doubt* OR disinform* OR misinform*) AND (scien* OR evidence OR *inform*)) AND TITLE-ABS-KEY ( climate OR "global warming" ) AND TITLE-ABS-KEY ( respon* OR counter* OR overcom* OR rebut* OR tackl* OR *bunk* OR communicat* OR refut* OR inoculat*) ) AND PUBYEAR < 2022 AND ( LIMIT-TO ( SRCTYPE,"j" ) ) AND ( LIMIT-TO ( DOCTYPE,"ar" ) OR LIMIT-TO ( DOCTYPE,"re" ) ) AND ( LIMIT-TO ( LANGUAGE,"English" ) )

**Step Two**: Title Scan Criteria

Each of the three co-authors independently went through the 620 titles of the found articles. They answered three criteria of selection, marking 1 each in response to the below questions:

- Does the article title mention climate change, global warming or similar?
- Does the article title mention denial, skepticism, doubt, misinformation or similar?
- Does the article title mention responding to, overcoming, correcting, reduction of- (and other alternatives)?

Authors were given the opportunity to make comments during title selection for clarification or notes.

**Step Three:** Titles marked with 8 or 9 points were automatically included for full read, as this would indicate at least two of the co-authors agreed that the article appeared to be relevant. This resulted in 22 articles for review.

**Step Four:** Titles marked with 5-7 points had their abstracts scanned to account for doubt over relevance, or difference in title scan interpretations. One author further commented with “abstract” to mark those texts with unclear titles for abstract scanning, another author scanned those indicated abstracts. A further 51 articles were included, resulting in a total of 73 articles for full read.

**Step Five:** A full read and analysis of texts according to a set of formulated questions by all authors led to a further rejection of 13, resulting in 61 articles to be included in the present study. Two texts were excluded for being an interview or viewpoint. A further eleven were excluded for not discussing, in a broad sense, interventions in order to reduce climate denial or induce belief in climate change.

**Stage Two**

**Step Six**: Upon review, it was suggested that the inclusion of various “counteraction terms” (i.e. respon*, counter* etc) in the search string may have excluded important studies. It would be more appropriate to find these papers without counteraction terms. The following search string was used to explore the entire literature on climate science denial:

( TITLE-ABS-KEY ( ( denial* OR deny OR skeptic* OR doubt* OR disinform* OR misinform*) AND (scien* OR evidence OR *inform*)) AND TITLE-ABS-KEY ( climate OR "global warming" ) ) AND PUBYEAR < 2022 AND ( LIMIT-TO ( SRCTYPE,"j" ) ) AND ( LIMIT-TO ( DOCTYPE,"ar" ) OR LIMIT-TO ( DOCTYPE,"re" ) ) AND ( LIMIT-TO ( LANGUAGE,"English" ) )

**Step Seven**: Title Scan Criteria

Of the 1354 articles identified, each of the three co-authors independently went through the 734 titles not found in step 1. They followed the same process as in step 1.

**Step Eight:** Step 3 was repeated, which resulted in 4 articles for review.

**Step Nine:** Step 4 was repeated, and a further 7 articles were included, resulting in a total of 11 articles for full read.

**Step Ten:** A full read and analysis of texts according to a set of formulated questions by all authors led to a further rejection of 4 for not being of relevance, resulting in 7 extra articles to be included in the study, adding to a grand total of 68 articles.

**Step Eleven:** A final double-check through all texts found that 3 were not peer-reviewed, each of which was removed at this stage, leaving 65 articles for the study.

**Full summary of selection process:**

Stage one found 620 articles in the search string, of which 22 titles were found to be of relevance and 51 abstracts indicated relevance. Upon full read 61 papers were included in the review. Stage two increased the search string to an extra 734 papers, of which 4 titles were found to be of relevance and 7 abstracts indicated relevance. Upon full read 7 extra articles were included. 3 were then excluded from the total list. The total number of papers under review in the present study is 65.

**Supplement B:** **List of 65 Articles reviewed within Section 4**

27 Empirical articles that test different interventions

Bain, P.G. *et al.* (2012) ‘Promoting pro-environmental action in climate change deniers’, *Nature Climate Change*, 2(8), pp. 600–603. Available at: https://doi.org/10.1038/nclimate1532.

Benegal, S.D. and Scruggs, L.A. (2018) ‘Correcting misinformation about climate change: the impact of partisanship in an experimental setting’, *Climatic Change*, 148(1), pp. 61–80. Available at: https://doi.org/10.1007/s10584-018-2192-4.

Bolsen, T., Palm, R. and Kingsland, J.T. (2019) ‘Counteracting Climate Science Politicization With Effective Frames and Imagery’, *Science Communication*, 41(2), pp. 147–171. Available at: https://doi.org/10.1177/1075547019834565.

Chinn, S. and Hart, P.S. (2021) ‘Effects of consensus messages and political ideology on climate change attitudes: inconsistent findings and the effect of a pretest’, *Climatic Change*, 167(3), p. 47. Available at: https://doi.org/10.1007/s10584-021-03200-2.

Cook, J., Lewandowsky, S. and Ecker, U.K.H. (2017) ‘Neutralizing misinformation through inoculation: Exposing misleading argumentation techniques reduces their influence’, *PLOS ONE*, 12(5), p. e0175799. Available at: https://doi.org/10.1371/journal.pone.0175799.

Dixon, G., Hmielowski, J. and Ma, Y. (2017) ‘Improving Climate Change Acceptance Among U.S. Conservatives Through Value-Based Message Targeting’, *Science Communication*, 39(4), pp. 520–534. Available at: https://doi.org/10.1177/1075547017715473.

Feygina, I., Jost, J.T. and Goldsmith, R.E. (2010) ‘System justification, the denial of global warming, and the possibility of “system-sanctioned change”’, *Personality & Social Psychology Bulletin*, 36(3), pp. 326–338. Available at: https://doi.org/10.1177/0146167209351435.

Franzen, A. and Mader, S. (2020) ‘Can Climate Skeptics Be Convinced? The Effect of Nature Videos on Environmental Concern’, *Sustainability*, 12(7), p. 2972. Available at: https://doi.org/10.3390/su12072972.

Gehlbach, H., Robinson, C.D. and Vriesema, C.C. (2019) ‘Leveraging cognitive consistency to nudge conservative climate change beliefs’, *Journal of Environmental Psychology*, 61, pp. 134–137. Available at: https://doi.org/10.1016/j.jenvp.2018.12.004.

Häkkinen, K. and Akrami, N. (2014) ‘Ideology and climate change denial’, *Personality and Individual Differences*, 70, pp. 62–65. Available at: https://doi.org/10.1016/j.paid.2014.06.030.

Hess, D.J. and Maki, A. (2019) ‘Climate change belief, sustainability education, and political values: Assessing the need for higher-education curriculum reform’, *Journal of Cleaner Production*, 228, pp. 1157–1166. Available at: https://doi.org/10.1016/j.jclepro.2019.04.291.

Hobson, K. and Niemeyer, S. (2013) ‘“What sceptics believe”: The effects of information and deliberation on climate change scepticism’, *Public Understanding of Science*, 22(4), pp. 396–412. Available at: https://doi.org/10.1177/0963662511430459.

Johnson, D.R. (2017) ‘Improving Skeptics’ Reasoning When Evaluating Climate Change Material: A Cognitive Intervention’, *Ecopsychology* [Preprint]. Available at: https://doi.org/10.1089/eco.2017.0012.

Joslyn, S.L. and LeClerc, J.E. (2016) ‘Climate Projections and Uncertainty Communication’, *Topics in Cognitive Science*, 8(1), pp. 222–241. Available at: https://doi.org/10.1111/tops.12177.

Lawrence, E.K. and Estow, S. (2017) ‘Responding to misinformation about climate change’, *Applied Environmental Education & Communication*, 16(2), pp. 117–128.

Maertens, R., Anseel, F. and van der Linden, S. (2020) ‘Combatting climate change misinformation: Evidence for longevity of inoculation and consensus messaging effects’, *Journal of Environmental Psychology*, 70, p. 101455. Available at: https://doi.org/10.1016/j.jenvp.2020.101455.

McCright, A.M. *et al.* (2016) ‘Examining the Effectiveness of Climate Change Frames in the Face of a Climate Change Denial Counter-Frame’, *Topics in Cognitive Science*, 8(1), pp. 76–97. Available at: https://doi.org/10.1111/tops.12171.

Motta, M., Ralston, R. and Spindel, J. (2021) ‘A Call to Arms for Climate Change? How Military Service Member Concern About Climate Change Can Inform Effective Climate Communication’, *Environmental Communication*, 15(1), pp. 85–98. Available at: https://doi.org/10.1080/17524032.2020.1799836.

Munoz-Carrier, G., Thomsen, D. and Pickering, G.J. (2020) ‘Psychological and experiential factors affecting climate change perception: learnings from a transnational empirical study and implications for framing climate-related flood events’, *Environmental Research Communications*, 2(4), p. 045003. Available at: https://doi.org/10.1088/2515-7620/ab89f9.

Porter, E., Wood, T.J. and Bahador, B. (2019) ‘Can presidential misinformation on climate change be corrected? Evidence from Internet and phone experiments’, *Research & Politics*, 6(3), p. 2053168019864784. Available at: https://doi.org/10.1177/2053168019864784.

Romero-Canyas, R. *et al.* (2019) ‘Bringing the Heat Home: Television Spots about Local Impacts Reduce Global Warming Denialism’, *Environmental Communication*, 13(6), pp. 740–760. Available at: https://doi.org/10.1080/17524032.2018.1455725.

Rotman, J.D., Weber, T.J. and Perkins, A.W. (2020) ‘Addressing Global Warming Denialism: The Efficacy of Mechanism-Based Explanations in Changing Global Warming Beliefs’, *Public Opinion Quarterly*, 84(1), pp. 74–103. Available at: https://doi.org/10.1093/poq/nfaa002.

Sauer, K.A. *et al.* (2021) ‘Six minutes to promote change: People, not facts, alter students’ perceptions on climate change’, *Ecology and Evolution*, 11(11), pp. 5790–5802. Available at: https://doi.org/10.1002/ece3.7553.

Stevenson, K.T. *et al.* (2014) ‘Overcoming skepticism with education: interacting influences of worldview and climate change knowledge on perceived climate change risk among adolescents’, *Climatic Change*, 126(3), pp. 293–304. Available at: https://doi.org/10.1007/s10584-014-1228-7.

Webb, B.S. and Hayhoe, D. (2017) ‘Assessing the Influence of an Educational Presentation on Climate Change Beliefs at an Evangelical Christian College’, *Journal of Geoscience Education*, 65(3), pp. 272–282. Available at: https://doi.org/10.5408/16-220.1.

Williams, M.N. and Bond, C.M.C. (2020) ‘A preregistered replication of “Inoculating the public against misinformation about climate change”’, *Journal of Environmental Psychology*, 70, p. 101456. Available at: https://doi.org/10.1016/j.jenvp.2020.101456.

Wong-Parodi, G. and Feygina, I. (2021) ‘Engaging People on Climate Change: The Role of Emotional Responses’, *Environmental Communication*, 15(5), pp. 571–593. Available at: https://doi.org/10.1080/17524032.2020.1871051.

27 Reviews, Overviews, and Perspectives

Compton, J. *et al.* (2021) ‘Inoculation theory in the post-truth era: Extant findings and new frontiers for contested science, misinformation, and conspiracy theories’, *Social and Personality Psychology Compass*, 15(6), p. e12602. Available at: https://doi.org/10.1111/spc3.12602.

Cook, J. (2017) ‘Understanding and countering climate science denial’, *Journal & Proceedings of the Royal Society of New South Wales*, 150(2), pp. 207–219.

Edvardsson Björnberg, K. *et al.* (2017) ‘Climate and environmental science denial: A review of the scientific literature published in 1990–2015’, *Journal of Cleaner Production*, 167, pp. 229–241. Available at: https://doi.org/10.1016/j.jclepro.2017.08.066.

Goodwin, J. and Dahlstrom, M.F. (2014) ‘Communication strategies for earning trust in climate change debates’, *WIREs Climate Change*, 5(1), pp. 151–160. Available at: https://doi.org/10.1002/wcc.262.

Hornsey, M.J. and Fielding, K.S. (2017) ‘Attitude roots and Jiu Jitsu persuasion: Understanding and overcoming the motivated rejection of science’, *The American Psychologist*, 72(5), pp. 459–473. Available at: https://doi.org/10.1037/a0040437.

Lewandowsky, S. (2021) ‘Liberty and the pursuit of science denial’, *Current Opinion in Behavioral Sciences*, 42, pp. 65–69. Available at: https://doi.org/10.1016/j.cobeha.2021.02.024.

McCaffrey, M.S. and Buhr, S.M. (2008) ‘Clarifying Climate Confusion: Addressing Systemic Holes, Cognitive Gaps, and Misconceptions Through Climate Literacy’, *Physical Geography*, 29(6), pp. 512–528. Available at: https://doi.org/10.2747/0272-3646.29.6.512.

Ranney, M.A. and Velautham, L. (2021) ‘Climate change cognition and education: given no silver bullet for denial, diverse information-hunks increase global warming acceptance’, *Current Opinion in Behavioral Sciences*, 42, pp. 139–146. Available at: https://doi.org/10.1016/j.cobeha.2021.08.001.

Rode, J.B. *et al.* (2021) ‘Influencing climate change attitudes in the United States: A systematic review and meta-analysis’, *Journal of Environmental Psychology*, 76, p. 101623. Available at: https://doi.org/10.1016/j.jenvp.2021.101623.

Treen, K.M. d’I., Williams, H.T.P. and O’Neill, S.J. (2020) ‘Online misinformation about climate change’, *WIREs Climate Change*, 11(5), p. e665. Available at: https://doi.org/10.1002/wcc.665.

Turnpenny, J.R. (2012) ‘Lessons from post-normal science for climate science-sceptic debates: Climate science-sceptic debates’, *Wiley Interdisciplinary Reviews: Climate Change*, 3(5), pp. 397–407. Available at: https://doi.org/10.1002/wcc.184.

Van Rensburg, W. (2015) ‘Climate Change Scepticism: A Conceptual Re-Evaluation’, *SAGE Open*, 5(2), p. 2158244015579723. Available at: https://doi.org/10.1177/2158244015579723.

Wong-Parodi, G. and Feygina, I. (2020) ‘Understanding and countering the motivated roots of climate change denial’, *Current Opinion in Environmental Sustainability*, 42, pp. 60–64. Available at: https://doi.org/10.1016/j.cosust.2019.11.008.

Bedford, D. (2010) ‘Agnotology as a Teaching Tool: Learning Climate Science by Studying Misinformation’, *Journal of Geography*, 109(4), pp. 159–165. Available at: https://doi.org/10.1080/00221341.2010.498121.

Cook, J., Ellerton, P. and Kinkead, D. (2018) ‘Deconstructing climate misinformation to identify reasoning errors’, *Environmental Research Letters*, 13(2), p. 024018. Available at: https://doi.org/10.1088/1748-9326/aaa49f.

Das, A. (2020) ‘Combatting Climate Change Denial’, *Resonance*, 25(7), pp. 933–945. Available at: https://doi.org/10.1007/s12045-020-1010-2.

Farrell, J., McConnell, K. and Brulle, R. (2019) ‘Evidence-based strategies to combat scientific misinformation’, *Nature Climate Change*, 9(3), pp. 191–195. Available at: https://doi.org/10.1038/s41558-018-0368-6.

Ferkany, M. (2015) ‘Is it Arrogant to Deny Climate Change or is it Arrogant to Say it is Arrogant? Understanding Arrogance and Cultivating Humility in Climate Change Discourse and Education’, *Environmental Values*, 24(6), pp. 705–724. Available at: https://doi.org/10.3197/096327115X14420732702572.

Haltinner, K. and Sarathchandra, D. (2018) ‘Climate change skepticism as a psychological coping strategy’, *Sociology Compass*, 12(6), p. e12586. Available at: https://doi.org/10.1111/soc4.12586.

Hansson, S.O. (2017) ‘Science denial as a form of pseudoscience’, *Studies in History and Philosophy of Science Part A*, 63, pp. 39–47. Available at: https://doi.org/10.1016/j.shpsa.2017.05.002.

Hansson, S.O. (2018) ‘Dealing with climate science denialism: experiences from confrontations with other forms of pseudoscience’, *Climate Policy*, 18(9), pp. 1094–1102. Available at: https://doi.org/10.1080/14693062.2017.1415197.

Leuschner, A. (2018) ‘Is it appropriate to “target” inappropriate dissent? on the normative consequences of climate skepticism’, *Synthese*, 195(3), pp. 1255–1271. Available at: https://doi.org/10.1007/s11229-016-1267-x.

Mason, S.E. (2020) ‘Climate Science Denial as Willful Hermeneutical Ignorance’, *Social Epistemology*, 34(5), pp. 469–477. Available at: https://doi.org/10.1080/02691728.2020.1739167.

Rudiak-Gould, P. (2013) ‘Cross-Cultural Insights into Climate Change Skepticism’, *Bulletin of the American Meteorological Society*, 94(11), pp. 1707–1713. Available at: https://doi.org/10.1175/BAMS-D-12-00129.1.

Shirley, B.J. (2021) ‘Post-Fact Fact Sheets: Dissociative Framing as a Strategy to Work Past Climate Change Denial’, *Technical Communication*, 68(2), pp. 41–60.

Sterman, J.D. (2011) ‘Communicating climate change risks in a skeptical world’, *Climatic Change*, 108(4), p. 811. Available at: https://doi.org/10.1007/s10584-011-0189-3.

Watts, E. (2019) ‘Teaching Climate Science to Increase Understanding & Receptivity’, *The American Biology Teacher*, 81(5), pp. 308–316. Available at: https://doi.org/10.1525/abt.2019.81.5.308.

11 Empirical papers not on counteraction, but with indications for counteraction

Bliuc, A.-M. *et al.* (2015) ‘Public division about climate change rooted in conflicting socio-political identities’, *Nature Climate Change*, 5(3), pp. 226–229. Available at: https://doi.org/10.1038/nclimate2507.

Busch, K.C. (2021) ‘Textbooks of doubt, tested: the effect of a denialist framing on adolescents’ certainty about climate change’, *Environmental Education Research*, 27(11), pp. 1574–1598. Available at: https://doi.org/10.1080/13504622.2021.1960954.

Capstick, S.B. and Pidgeon, N.F. (2014) ‘What is climate change scepticism? Examination of the concept using a mixed methods study of the UK public’, *Global Environmental Change*, 24, pp. 389–401. Available at: https://doi.org/10.1016/j.gloenvcha.2013.08.012.

Chen, C.-F. *et al.* (2021) ‘Social bots’ role in climate change discussion on Twitter: Measuring standpoints, topics, and interaction strategies’, *Advances in Climate Change Research*, 12(6), pp. 913–923. Available at: https://doi.org/10.1016/j.accre.2021.09.011.

Harvey, J.A. *et al.* (2018) ‘Internet Blogs, Polar Bears, and Climate-Change Denial by Proxy’, *BioScience*, 68(4), pp. 281–287. Available at: https://doi.org/10.1093/biosci/bix133.

Lewandowsky, S. *et al.* (2015) ‘Seepage: Climate change denial and its effect on the scientific community’, *Global Environmental Change*, 33, pp. 1–13. Available at: https://doi.org/10.1016/j.gloenvcha.2015.02.013.

Lo, A.Y. (2014) ‘The right to doubt: climate-change scepticism and asserted rights to private property’, *Environmental Politics*, 23(4), pp. 549–569. Available at: https://doi.org/10.1080/09644016.2014.884310.

Sarathchandra, D. and Haltinner, K. (2020) ‘Trust/distrust judgments and perceptions of climate science: A research note on skeptics’ rationalizations’, *Public Understanding of Science (Bristol, England)*, 29(1), pp. 53–60. Available at: https://doi.org/10.1177/0963662519886089.

Sezen-Barrie, A., Shea, N. and Borman, J.H. (2019) ‘Probing into the sources of ignorance: science teachers’ practices of constructing arguments or rebuttals to denialism of climate change’, *Environmental Education Research*, 25(6), pp. 846–866. Available at: https://doi.org/10.1080/13504622.2017.1330949.

Trémolière, B. and Djeriouat, H. (2021) ‘Exploring the roles of analytic cognitive style, climate science literacy, illusion of knowledge, and political orientation in climate change skepticism’, *Journal of Environmental Psychology*, 74, p. 101561. Available at: https://doi.org/10.1016/j.jenvp.2021.101561.

Van Rensburg, W. and Head, B.W. (2017) ‘Climate Change Scepticism: Reconsidering How to Respond to Core Criticisms of Climate Science and Policy’, *SAGE Open*, 7(4), p. 2158244017748983. Available at: https://doi.org/10.1177/2158244017748983.
